# Wildlife Waterfowl as a Source of Pathogenic *Campylobacter* Strains

**DOI:** 10.3390/pathogens11020113

**Published:** 2022-01-18

**Authors:** Beata Wysok, Marta Sołtysiuk, Tomasz Stenzel

**Affiliations:** 1 Department of Veterinary Public Health, Faculty of Veterinary Medicine, University of Warmia and Mazury in Olsztyn, 10-719 Olsztyn, Poland; beata.wysok@uwm.edu.pl (B.W.); marta.soltysiuk@uwm.edu.pl (M.S.); 2 Department of Poultry Diseases, Faculty of Veterinary Medicine, University of Warmia and Mazury in Olsztyn, 10-719 Olsztyn, Poland

**Keywords:** *Campylobacter*, antimicrobial resistance, virulence genes, game species, wildlife waterfowl

## Abstract

Background: The aim of the study was to determine whether free-living birds belonging to game species whose meat is used for human consumption can constitute a reservoir of pathogenic *Campylobacter* strains, spreading these bacteria to other hosts or directly contributing to human infection. Methods: A total of 91 cloacal swabs were taken from different species of wildlife waterfowl to estimate the *Campylobacter* prevalence, the genetic diversity of the isolates, and the presence of virulence genes and to evaluate the antimicrobial resistance. Results: The presence of *Campylobacter* spp. was confirmed in 32.9% of samples. Based on *flaA*-SVR sequencing, a total of 19 different alleles among the tested *Campylobacter* isolates were revealed. The virulence genes involved in adhesion were detected at high frequencies among *Campylobacter* isolates regardless of the host species. The highest resistance was observed for ciprofloxacin. The resistance rates to erythromycin and tetracycline were observed at the same level. Conclusions: These results suggest that wildlife waterfowl belonging to game species may constitute a reservoir of *Campylobacter,* spreading these bacteria to other hosts or directly contributing to human disease. The high distribution of virulence-associated genes among wildlife waterfowl *Campylobacter* isolates make them potentially able to induce infection in humans.

## 1. Introduction

Free-living birds, including migratory species, can become vectors for a wide range of microorganisms that can be transmissible to other animals and humans [1]. In addition, bird migration provides a mechanism for the establishment of new endemic foci of disease at great distances from where an infection was acquired [2]. The intestinal tract of birds may be colonized by different bacteria, many of which are pathogenic for humans. Moreover, the close association of birds and humans in urban and agricultural settings facilitates zoonotic disease transfer [3,4]. Although bird infestations may be transmitted to other animals and humans via direct contact or inhalation of contaminated air conditioners or vents, the most common is oral transmission through food and water that has been contaminated by bird fecal material [5]. A leading worldwide foodborne zoonosis is campylobacteriosis [6]. *Campylobacter* spp. commonly inhabit the intestines of avian species, as their body temperature provides an optimal environment for the growth of the organism. Therefore, these bacteria are found in both poultry and wild bird feces [7,8]. Moreover, some wild bird species have successfully adapted to anthropogenic environments and routinely come into close contact with livestock, domestic animals, and people and are thus seen as a potential source of *Campylobacter* [9]. It is also important that numerous wildlife birds are game species whose meat is used for human consumption and can pose a potential health hazard [10]. For a better understanding of the epidemiology and transmission of *Campylobacter* spp., an investigation of the genetic relatedness of *Campylobacter* isolates is crucial. Many molecular methods have been developed to investigate the diversity within *Campylobacter* isolates, including a sequence analysis of the short variable region (SVR) of the *flaA* gene. This highly discriminatory method is widely used for a better understanding of *Campylobacter* population structures [11,12]. According to Hanage [13], in most pathogenic bacteria, the population is made up of multiple distinct lineages. which are associated with properties such as virulence or drug resistance. In the case of campylobacteriosis, both successful invasion and organization in host cells depend on various virulence factors linked with adhesion to intestinal mucosa, invasion of epithelial cells, toxin production, and protein secretion [14]. Among adhesion-associated markers, the following are crucial: the *flaA* gene, encoding the major flagellin protein (FlaA), a structural component of flagella crucial for attachment to intestinal epithelial cells and involved in autoagglutination and microcolony formation [15,16]; the *cadF* gene, encoding a fibronectin binding protein CadF [17]; the *racR* gene, encoding a DNA-binding response regulator [18]; a periplasmic cytochrome C peroxidase, encoded by *docA*; and the chaperone protein DnaJ, encoded by the *dnaJ* gene [19]. Regarding markers affecting invasion, a significant role is played by the *pldA* gene, encoding phospholipase A; the *ciaB* gene, encoding a *Campylobacter* invasion antigen; the *virB11* gene, responsible for host cell invasion; and invasion-associated marker (*iam*) [20]. In addition, numerous toxins produced by *Campylobacter* spp. have been described, but only cytolethal distending toxin (CDT), encoded by three linked genes, namely *cdtA*, *cdtB*, and *cdtC*, has been well characterized [21]. *Campylobacter* infection in humans commonly causes gastroenteritis, but infection can also occur outside the intestines, such as polyneuropathic disorder, denominated as Guillain-Barré syndrome (GBS). The *Campylobacter* strains that can elicit GBS carry either *wlaN* or *cgtB*, both encoding a β-1,3-galactosyltransferase enzyme that is required for the production of sialylated lipooligosaccharide LOS^SIAL^, a crucial virulence factor of GBS [22].

For a better understanding of the evolution of infectious diseases, the determination of bacteria drug resistance is crucial. According to Bonnedahl and Järhult [23], wild birds should be postulated not only as reservoirs but also as potential spreaders of antibiotic resistance. Among the factors contributing to the prevalence of antibiotic resistance among wild birds, the natural preservation state, livestock, human densities, and the remoteness of an area have a significant impact [24]. Moreover, according to Skurnik et al. [25] and Allen et al. [24], the levels of resistance seem to correlate with the degree of proximity to human settlements. As wild birds seem to play a significant role as reservoirs for pathogenic enteric bacteria, they can pollute the environment with antimicrobial-resistant (AMR) bacteria and spread difficult-to-treat zoonotic diseases. 

This study aimed to better understand the role of wildlife waterfowl in the transmission of *Campylobacter* infection among both livestock and humans. This study aimed specifically to determine (i) the prevalence rate of *Campylobacter* in wildlife waterfowl belonging to game species, (ii) the genetic diversity, (iii) the prevalence of virulence genes related to adherence, invasion cytotoxicity and GBS, as well as the antibiotic resistance profile in the investigated *Campylobacter* isolates.

## 2. Results

### 2.1. Isolation and Identification of Bacterial Strains

Out of 91 tested cloacal swabs, the presence of *Campylobacter* spp. was confirmed in 30 (32.9%) samples. The prevalence rate ranged from 45.5% among white-fronted geese (in 5 out of 11) to 32.8% among mallards (in 20 out of 61). None of the fecal samples from Eurasian teal were positive for *Campylobacter* spp. (Table 1). The majority of the obtained isolates (28/30, 93.3%) were *C. jejuni*, and only two (6.7%) isolates from mallards were *C. coli.*

### 2.2. Detection of Virulence Genes 

**Figure 1:**
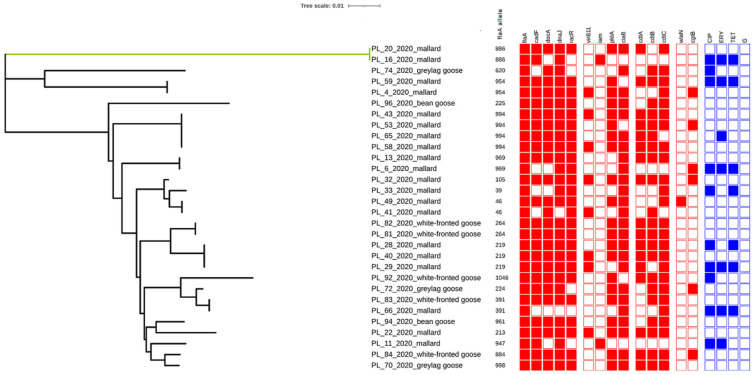
Maximum likelihood tree of *Campylobacter flaA*-SVR allele sequences among isolates originating from wild birds. For each isolate, the following characteristics are shown: strain ID (according to the pattern: country of isolation_individual number of tested sample_year of isolation_host), *flaA* allele number, virulence genes, and antimicrobial resistance. The prevalence of determinants involved in virulence is indicated by red (present) and white (absent) squares. The occurrence of resistance to tested antimicrobials is indicated by blue (present) and white (absent) squares. *C. coli* isolates form the green cluster. The figure is visualized in the interactive tree of life (iTol).

The virulence genes involved in adhesion were detected at high frequencies among *Campylobacter* isolates regardless of the source. All isolates originating from white-fronted geese and bean geese possessed *flaA*, *cadF*, *racR*, *docA*, and *dnaJ* genes. Moreover, high frequency rates were noted for these genes among isolates from mallards (100%, 80%, 85%, 75%, and 90%) and graylag geese (100%, 66.7%, 66.7%, 66.7%, and 100%, respectively). Regarding genes associated with invasion, the most prevailing were the *ciaB* gene (with prevalence rates ranging from 100% in bean geese and greylag geese to 80% in mallards and white-fronted geese) and the *pldA* gene (with prevalence rates ranging from 100% in bean geese and white-fronted geese to 60% in mallards). The *virB11* and *iam* genes were noted only among mallard-origin isolates at the level of 40% (8/20) and 10% (2/20), respectively. Among genes associated with cytotoxicity, the most common were *cdtB* and *cdtC* genes, noted in 100% of isolates from white-fronted geese, graylag geese, and bean geese and in 55% (11/20) and 75% (15/20) of mallard-origin isolates. However, the *cdtA* gene was detected in 60% (12/20), 80% (4/5), and 33.3% (1/3) of *Campylobacter* strains isolated from mallards, white-fronted geese, and greylag geese, respectively. The cytotoxin-encoding cluster *cdtABC* was confirmed in 9 out 20 (45%) of mallard isolates, in four out of five (80%) of white-fronted geese isolates, and in one out of three (33.3%) greylag geese isolates. Only single isolates possessed LOS^SIAL^-related genes, 5% (1/20) of mallard-origin isolates were positive for the *wlaN* gene, and 20% (4/20), 20% (1/5), and 33.3% (1/3) of isolates recovered from mallards, white-fronted geese, and graylag geese were positive for the *cgtB* gene (Figure 1). 

The results showed that no *Campylobacter* strain was positive for all tested virulence markers, while none of the analyzed strains showed a lack of all tested genes.

### 2.3. Sequencing of flaA-SVR

The conducted *flaA*-SVR sequencing revealed a total of 19 different alleles among 30 tested *Campylobacter* isolates (Figure 1). The highest divergence was observed among isolates originating from greylag and bean geese, with a Simpson’s diversity index of 1.0. However, among mallards and white-fronted geese, this index was 0.932 (CI95% 0.884–0.979) and 0.9 (CI 95% 0.725–1.000), respectively.

The most commonly reported *flaA*-SVR alleles were 994 and 219, which were only noted among isolates originating from mallards and covering 23.3% of obtained isolates. Eleven out of 19 (57.9%) alleles occurred only once. Only the *flaA*-SVR allele 391 was not specific to the host and was noted among *Campylobacter* strains isolated from mallards and white-fronted geese.

### 2.4. Antimicrobial Resistance

Among the four tested antimicrobial agents, the highest resistance was observed for ciprofloxacin (in 10 out of 30 isolates, 33.3%) (Figure 1). The majority of ciprofloxacin-resistant isolates were isolated from mallards (8 out of 10, 80%). The remaining isolates originated from graylag goose (one isolate) and white-fronted goose (one isolate). The resistance rates to erythromycin and tetracycline were observed at the same level of 23.3% (in 7 out of 30 isolates), and the resistant isolates were only recovered from mallards. All *Campylobacter* isolates, regardless of the source, did not show resistance to gentamicin. 

The most frequent resistance pattern was CIP_ERY_TET, noted in 5 out 30 (16.7%) tested isolates. None of the isolates obtained manifested resistance to the four tested antimicrobial agents. Simultaneously, sensitivity to all tested antimicrobials was observed in the majority of tested isolates with an overall rate of 63.3% (100% of bean geese, 80% of white-fronted geese, 66.7% of graylag geese, and 55% of mallards).

## 3. Discussion

Since campylobacteriosis has become considered an emerging foodborne disease in recent years, the majority of studies have concentrated on determining the source of *Campylobacter* among farm animals. However, wildlife waterfowl may play a role in the spread of campylobacteriosis through fecal contamination of the environment, feed, and surface water. Therefore, Elmberg et al. [26] emphasized the application of precautionary principles to ensure that domestic poultry does not come into contact (or share pasture or water access) with wild birds. Moreover, wildlife waterfowl may pose a risk not only to other animals but also to humans due to direct contact with birds or their feces in beaches or parks or via the consumption of vegetables infected by their feces, at least via consumption of undercooked meat from wildlife waterfowl. Wildlife waterfowl game species can also be a source of direct infection, especially when their bodies are hit by numerous pellets, which can damage the intestines and can contaminate meat with the intestinal content. 

French et al. [27] suggest that feces from wildlife waterfowl in playgrounds could contribute to the occurrence of campylobacteriosis in preschool children. According to Ramonaite et al. [28], the prevalence of *Campylobacter* spp. among wildlife waterfowl varied from 1.4% to 72.7% depending on different countries and wild bird species. In the present study, 32.9% of wildlife waterfowl tested were positive for *Campylobacter* spp. The predominant species associated with human illness is *Campylobacter jejuni*, also described as dominant in poultry in different geographical regions [29]. Additionally, in the current study, 93.3% of isolates originating from examined birds were identified as *C. jejuni.* Moreover, *Campylobacter* spp. is described as genetically divergent, which is in accordance with the current study. The overall Simpson’s diversity index calculated for all 30 *Campylobacter* isolates originating from wildlife waterfowl was estimated at a value of 0.966. All *flaA*-SVR alleles assigned to greylag geese and bean geese isolates occurred only once, while among isolates obtained from white-fronted geese, single sequences only occurred twice. This is in contrast with the results obtained in previous studies [30], which reported that the majority of alleles co-existed among poultry (18/28, 64.3%) and human (22/34, 64.7%) isolates. According to Atterby et al. [31], strain-specific association to particular bird species is noticeable, and limited contact between wildlife waterfowl species differences in diet or feeding behavior or migration patterns can be seen as the causes of the described situation. Interestingly, studies performed by Colles et al. [32] on *Campylobacter* populations in wild and domesticated Mallard ducks revealed that only one sequence type was shared between the two sources, accounting for 0.9% of wild duck isolates and 5% of farmed duck isolates. In the present study, it was also noted that only a single allele was not unique to the source-*flaA*-SVR allele 391 was noted among mallards and white-fronted geese. An analysis of sequences deposited in the PubMLST database revealed that only a few *flaA*-SVR alleles (alleles 8, 219, 886, 46, 264) were found in *Campylobacter* strains isolated from environmental water samples, chicken meat, offal, or from human stool. Moreover, studies performed by Di Giannatale et al. [33] and Llarena et al. [34] revealed several genotypes overlapping in wild birds, farm animals, poultry, and human isolates. 

For a better understanding of the epidemiology of *Campylobacter* infection, it is crucial not only to recognize the various sources of this pathogen but also to determine the virulence properties of bacteria since different pathogenic profiles can be identified within the species [35]. The current results have revealed the common prevalence of genes associated with adhesion (*flaA*, *cadF*, *racR*, *docA*, and *dnaJ*) among isolates of wildlife waterfowl origin, which is significant since every *Campylobacter* infection is preceded by colonization of the intestinal tract. Similar results have been previously noted by Du et al. [36] in China, Wei et al. [37] in South Korea, and Shyaka et al. [38] in Japan. These findings confirmed the strong colonization ability among strains isolated from wildlife waterfowl. Generally, the prevalence of genes involved in adhesion is common among isolates, regardless of the source and geographical region [39,40]. Regarding invasion abilities, the authors’ previous studies have revealed the common prevalence of *ciaB* (83.3%) and *pldA* (70%) genes among wildlife waterfowl, regardless of the source. The high prevalence of these genes was previously noted in wild bird isolates [37], layer poultry isolates [41], chicken meat isolates [42], and in human and cattle isolates [43]. The overall prevalence rates of two other tested virulence genes associated with invasion (*virB11* and *iam*) were 26.7% and 6.7%, respectively. The low percentage of *virB11*- and *iam*-positive *Campylobacter* isolates was also detected in retail chicken meat in China [44], in Danish pigs and cattle [45], as well as in human isolates in Chile [46]. Some authors have also noted that the role of *virB11* gene in pathogenesis of campylobacteriosis is still not clear [37] although Tracz et al. [47] suggested that products of pVir plasmid genes may effect a more serious course of *Campylobacter* infection in humans, resulting in bloody diarrhea. Similar divergent observations were described in relation to the invasion-associated marker *iam*. Sanad et al. [48] suggested that the use of the *iam* as a virulence determinant in epidemiological studies might be potentially misleading and might require reevaluation. 

One of the main virulence factors related to *Campylobacter* spp. is cytolethal-distending toxin (CDT), encoded by the three adjacent genes *cdtA*, *cdtB,* and *cdtC* [49]. The carriage of *cdt* complex is common in isolates from poultry [49], swine [45], cattle [50], and human [30,51] isolates. In the present study, it was found that 46.7% of wildlife waterfowl isolates possessed three *cdt* genes. 

As *Campylobacter* is known as one of the main etiological factors connected with the GBS occurrence in humans, it appears crucial to establish the occurrence of pathogenic genes involved in this process. It is known that *Campylobacter* strains carrying *wlaN* and *cgtB* genes responsible for LOS^SIAL^ production can potentially elicit GBS due to the well-documented molecular mimicry between the LOS^SIAL^ and the saccharide component of the human GM1 ganglioside, which is present in peripheral nerves [52]. A differential distribution of the *cgtB* and *wlaN* genes among wildlife waterfowl was noted. Only single isolates originating from wildlife waterfowl carried *wlaN* (3.3% of isolates) or *cgtB* (20% of isolates) genes. The prevalence rates of *wlaN* and *cgtB* genes were noted at similar levels in previous studies. These genes were detected in 11.3% of wildlife waterfowl isolates in South Korea [37], in 25% of human isolates in Japan [53], in 17.5% of geese carcasses in Poland [54], in 6.7% of human isolates in Argentina [55], and up to 21.9% of livestock animals in Spain [56]. 

In recent years, antibiotic resistance among pathogenic bacteria has become an emerging problem. The widespread use of antibiotics in industrial agriculture, mainly in animal production, has contributed to the threat of drug resistance, as the resistant bacteria in animals may directly or indirectly reach humans through food or water [57]. The increasing rates of resistance of *Campylobacter* isolates to fluoroquinolones and macrolides observed in recent years pose a significant risk for human health since these antimicrobial factors are commonly used in the treatment of *Campylobacter* infection [58]. Moreover, a significantly high percentage of tetracycline-resistant *Campylobacter* isolates has also been noted, which is alarming since tetracycline has been suggested as an alternative treatment choice for *Campylobacter* infection. In this study, the overall resistance rates to ciprofloxacin, erythromycin and tetracycline among *Campylobacter* isolates of wildlife waterfowl origin were 33.3%, 23.3%, and 23.3%, respectively. Interestingly, these rates were mostly mediated by isolates originating from mallards. Studies performed by Du et al. [36] in China on wildlife waterfowl from different locations and sites showed that wildlife waterfowl from urban areas have higher antibiotic resistance compared to birds from suburban areas, which might be due to contaminated environment water. These findings are in accordance with the current results since mallards, in contrast to bean goose or white-fronted goose, settle more frequently in areas of human activities, which results in different resistance levels of *Campylobacter* isolates obtained from wildlife waterfowl. Interestingly, the observed rates of resistance were lower than those noted among isolates of farm animals and of human origin. The previous studies revealed that 74% of *Campylobacter* spp. isolated from livestock, poultry processing plants, and retail meat in North Carolina were resistant to tetracycline [59], and 76% and 64% of human isolates in the study performed in Italy were resistant to ciprofloxacin and tetracycline [60]. Similar findings were noted by Marotta et al. [61], who noted that compared with farmed poultry, the incidence of AMR in the *C. jejuni* isolates from the other bird groups was low, confirming that the poultry are much more exposed to antimicrobials.

The current results suggest that wildlife waterfowl belonging to game species may constitute a reservoir of *Campylobacter* spreading these bacteria to other hosts or directly contributing to human diseases. The high distribution of virulence-associated genes among wildlife waterfowl *Campylobacter* isolates makes them potentially able to induce infection in humans.

## 4. Materials and Methods

### 4.1. Isolation and Identification of Bacterial Strains

In this study, a total of 91 samples of cloacal swabs from wildlife waterfowl—61 from mallard ducks (*Anas platyrhynchos*), 11 from white-fronted geese (*Anser albifrons*), eight from greylag geese (*Anser anser*), six from Eurasian teal (*Anas crecca*), and five from bean geese (*Anser fabalis*)—were analyzed for the presence of *Campylobacter* spp. The samples were taken from birds hunted mainly in northeastern Poland between August and November 2020 (Supplementary Table S1). The swabs were transported to the laboratory in Amies gel transport medium (Oxoid, Basingstoke, UK) and were subsequently transferred to 9 mL of Bolton broth (Oxoid, UK). The enrichment cultures were grown at 37 °C for 4 h and then at 41.5 °C for 44 ± 4 h under microaerobic conditions (5% O_2_, 10% CO_2_, and 85% N). A loopful of the suspension was then spread on the surface of a charcoal cefoperazone deoxycholate modified agar (mCCDA, Oxoid, UK) and agar Karmali (Oxoid, UK). After incubation under microaerobic conditions for 24–48 h, the plates were examined for morphologically typical *Campylobacter* colonies. Single colonies were picked up and confirmed as *Campylobacter* by examination of microscopic morphology, the presence of oxidase activity, motility, and lack of microaerobic growth at 25 °C. Subsequently, the isolates were subcultured only once in order to minimize changes resulting from several passages and stored at −80 °C in defibrinated horse blood (Oxoid, UK) with added glycerol (80:20 *v*/*v*).

Species identification of the isolates was carried out based on primers listed in Table 2. For this purpose, *Campylobacter* isolates cultured on Columbia agar supplemented with blood were suspended in 1 mL of sterile water and centrifuged at 13,000× *g* for 1 min. The precipitate was suspended in a Tris buffer. DNA isolation was performed using Genomic -Mini Kit (A&A Biotechnology, Gdańsk, Poland) according to the manufacturer’s instructions. The purity and concentration of the DNA were determined spectrophotometrically. The DNA was used as a template in all the PCR assays (described in detail below).

### 4.2. Detection of Virulence Genes 

The genomic DNA was amplified by PCR to confirm the presence of genes involved in adherence (*flaA*, *cadF*, *docA*, *dnaJ*, and *racR*) and invasion (*virB11*, *iam*, *ciaB*, and *pldA*), responsible for the production of cytolethal distending toxin (*cdtA*, *cdtB*, and *cdtC*) and sialylated lipooligosaccharide (*wlaN* and *cgtB*) by using primers listed in Table 2. Amplification was performed in a 50-μL reaction mixture containing 5 μL of the PCR buffer (10 -times concentrated), 5 μL of dNTPs (final concentration of 200 µM), 0.5 μL of each primer (final concentration of 0.1 µM), 10 μL MgCl_2_ (final concentration of 5 mM), 2 μL (2 U) thermostable Taq polymerase (Thermo Fisher Scientific, Waltham, MA, USA), 5 μL of template DNA at the final concentration of 120 ng verified by Nano-DropTM Spectrophotometer (Thermo Fisher Scientific, USA), and DNase-and RNase-free deionized water. All PCRs were carried out using the following conditions: initial denaturation at 94 °C for 5 min followed by 30 cycles of denaturation for 1 min at 95 °C, annealing at a temperature specific to the primer pair for 1 min, and extension for 1 min at 72 °C. The final elongation step was carried out at 72 °C for 5 min. A positive control consisting of DNA extracted from *C. jejuni* ATCC 33291 and *C. coli* ATCC 43478 as well as a non-template PCR control consisting of PCR-grade water were included in each PCR run. The PCR product was run on a 2% agarose gel stained with ethidium bromide at a concentration of 5 μg/mL. The size of the amplification product was determined using the 100-bp molecular weight marker.F

### 4.3. Sequencing of flaA-SVR

The DNA of all isolates obtained in this study was subjected to *flaA* short variable region (SVR) and sequencing using the primers listed in Table 2. For PCR, the conditions were as described above. The PCR products were visualized in gel electrophoresis, purified with a Clean-Up Kit (A&A Biotechnology, Poland), and sequenced by Sanger sequencing (Genomed, Warszawa, Poland). The forward and reverse sequences were assembled using the Contig Express module in Vector NTI Express (Thermo Fisher Scientific, USA) and trimmed to a 321-bp length covering the *flaA*-SVR. The sequences were assigned *flaA*-SVR allele numbers according to the PubMLST database (http://pubmlst.org/campylobacter (accessed on 1 December 2021)), and a cluster analysis was then performed using default parameters in MEGA X v. 10.1 (http://www.megasoftware.net (accessed on 1 December 2021)). The maximum likelihood tree based on the *flaA*-SVR sequences was visualized in iTOL v4 (https://itol.embl.de (accessed on 1 December 2021)). The obtained sequences were submitted to the GenBank database and received the following Accession Numbers OL314289–OL314318.

The genetic diversity of *Campylobacter* isolates originating from wildlife waterfowl was assessed by the Simpson’s diversity index (ID) as described previously [69] using the online tool “Comparing Partitions” from the website http://www.comparingpartitions.info (accessed on 1 November 2021) [70].

### 4.4. Antimicrobial Resistance

Antimicrobial resistance was examined by the diffusion-disk method according to the protocol of the European Committee on Antimicrobial Susceptibility Testing (EUCAST) for fastidious organisms. All *Campylobacter* isolates were suspended in a brain-heart infusion (BHI) broth to a turbidity equivalent to a 0.5 McFarland standard. Mueller–Hinton agar plates supplemented with 5% of defibrinated horse blood (Oxoid, UK), and 20 mg/L of β-Nicotinamide Adenine Dinucleotide (β-NAD) (Sigma Aldrich, Saint Louis, MO, USA) were inoculated with the prepared suspension. The selected antimicrobials were in agreement with EUCAST recommendations as crucial in the treatment of *Campylobacter* infection. The following antibiotic disks were placed on the surface of the dry plates: erythromycin (ERY, 15 µg), gentamicin (GEN, 10 µg), ciprofloxacin (CIP, 5 µg), and tetracycline (TET, 30 µg). The plates were incubated at 41 ± 1 °C for 24–48 h in a microaerophilic atmosphere. Zones of inhibited growth for erythromycin, ciprofloxacin and tetracycline were determined according to EUCAST breakpoints [71] and The Clinical and Laboratory Standards Institute [72] breakpoints were used for gentamicin. The results were interpreted as resistant or sensitive. The inhibition zone readings defined as intermediate were classified as resistant. The strains that showed resistance to no less than three antimicrobial classes were considered multidrug-resistant (MDR) [73].

### 4.5. Statistical Analysis

Statistical tests were performed using Statistica (StatSoft, version 13.3, Poland). The analyses of the presence of virulence genes and antibiotic resistance profiles were performed using a non-parametric Kruskal–Wallis one-way analysis of variance followed by a non-parametric U Mann–Whitney test for pairwise comparisons. *p*-Values < 0.05 were considered significant.

## Figures and Tables

**Table 1:** Prevalence of *Campylobacter* spp. among wild birds.

Source		No. of Samples	No. of Positive Samples (%)	
Common Name	Latin Name		*C. jejuni*	*C. coli*
Mallard duck	*Anas platyrhynchos*	61	18 (29.5%)	2 (3.3%)
White-fronted goose	*Anser albifrons*	11	5 (45.5%)	0
Greylag goose	*Anser anser*	8	3 (37.5%)	0
Eurasian teal	*Anas crecca*	6	0	0
Bean goose	*Anser fabalis*	5	2 (40%)	0

**Table 2:** PCR primers used in the study.

Target Gene	Sequences (5′–3′)	Product Size (bp)	Annealing Temperature °C	References
*16S rRNA* for *Campylobacter* spp.	F-ATCTAATGGCTTAACCATTAAAC	857	58	[62]
	R GGACGGTAACTAGTTTAGTATT			
*mapA* for *C. jejuni*	F-CTATTTTATTTTTGAGTGCTTGTG	589	58	[62]
	R-GCTTTATTTGCCATTTGTTTTATTA			
*ceuE* for *C. coli*	F-AATTGAAAATTGCTCCAACTATG	462	58	[62]
	R-TGATTTTATTATTTGTAGCAGCG			
*flaA-SVR*	F-CTA TGG ATG AGC AAT T(AT)A AAA T	383	53	[63]
	R-CAA G(AT)C CTG TTC C(AT)A CTG AAG			
*flaA*	F-AATAAAAATGCTGATAAAACAGGTG	855	53	[53]
	R-TACCGAACCAATGTCTGCTCTGATT			
*flhA*	F-GGAAGCGGCACTTGGTTTGC	735	53	[64]
	R-GCTGTGAGTGAGATTATAGCAG			
*dnaJ*	F-ATTGATTTTGCTGCGGGTAG	177	50	[65]
	R-ATCCGCAAAAGCTTCAAAAA			
*cadF*	F-TTGAAGGTAATTTAGATATG	400	45	[66]
	R-CTAATACCTAAAGTTGAAAC			
*virB11*	F-TCTTGTGAGTTGCCTTACCCCTTTT	494	53	[53]
	R-CCTGCGTGTCCTGTGTTATTTACCC			
*docA*	F-ATAAGGTGCGGTTTTGGC	725	50	[64]
	R-GTCTTTGCAGTAGATATG			
*iam*	F-GCGCAAAATATTATCACCC	518	52	[67]
	R-TTCACGACTACTATGCGG			
*ciaB*	F-TGCGAGATTTTTCGAGAATG	527	54	[65]
	R-TGCCCGCCTTAGAACTTACA			
*racR*	F-GATGATCCTGACTTTG	584	45	[53]
	R-TCTCCTATTTTTACCC			
*pldA*	F-AAGCTTATGCGTTTTT	913	45	[53]
	R-TATAAGGCTTTCTCCA			
*cdtA*	F-CCTTGTGATGCAAGCAATC	370	49	[53]
	R-ACACTCCATTTGCTTTCTG			
*cdtB*	F-CAGAAAGCAAATGGAGTGTT	620	51	[53]
	R-AGCTAAAAGCGGTGGAGTAT			
*cdtC*	F-CGATGAGTTAAAACAAAAAGATA	182	47	[53]
	R-TTGGCATTATAGAAAATACAGTT			
*wlaN*	F-TGCTGGGTATACAAAGGTTGTG	330	55	[64]
	R-ATTTTGGATATGGGTGGGG			
*cgtB*	F-TAAGAGCAAGATATGAAGGTG	561	52	[68]
	R-GCACATAGAGAACGCTACAA			

## Data Availability

The data presented in this study are available on request from the corresponding author. The data are not publicly available, as they are still used for other research works.

## Supplementary Materials

The following supporting information can be downloaded at: https://www.mdpi.com/article/10.3390/pathogens11020113/s1, Table S1: The description of *Campylobacter* spp. isolates obtained from wildlife waterfowl.
